# *Dorstenia luamensis* (Moraceae), a new species from eastern Democratic Republic of Congo

**DOI:** 10.3897/phytokeys.42.7604

**Published:** 2014-10-24

**Authors:** Miguel E. Leal

**Affiliations:** 1 Wildlife Conservation Society, Kiwafu Road 802, Kansanga, Kampala, Uganda

**Keywords:** *Dorstenia*, Albertine Rift, endemic, lithophytic

## Abstract

A new species of *Dorstenia* L. (*Moraceae*), *Dorstenia luamensis* M.E.Leal, is described from the Luama Wildlife Reserve, west of Lake Tanganyika and north of the town of Kalemie in the eastern part of the Democratic Republic of Congo (DRC). This species is endemic to the region and differs from any of the other species by its fernlike lithophytic habit and lack of latex. A description and illustration of this species is presented here. *Dorstenia luamensis* M.E.Leal inhabits moist and shady vertical rock faces close to small waterfalls in the forest; the species is distributed in small populations within the type locality, and merits the conservation status of endangered (EN).

## Introduction

In 2012, a specimen of *Dorstenia* L. was collected from the Luama Wildlife Reserve in eastern DRC (M.E. Leal 2551) hanging from vertical cliffs and rock faces close to a waterfall on the shear zone of two figaus. These plants have a typical *Dorstenia* L. inflorescence but their hanging habit and lack of latex is unusual. The only other hanging *Dorstenia* species (hemi-epiphyte) is *Dorstenia astyanactis* Aké Assi first described from Ivory Coast (Ake Assi 1967) and later also collected in Cameroon (Pollard et al. 2003). Its inflorescence only has one appendix whereas the specimens from Luama have between 8 to12 appendices. The only other species in Africa mentioned in the revision of Berg and Hijmans (1999) growing on rocks is *Dorstenia zanzibarica* Oliver, but this species has dentate leaves and its receptacle is triangular to subquadrangular.

The specimens collected in the Luama Wildlife Reserve keyed out to the section of *Kosaria* using Berg and Hijman (1999) key to the sections of *Dorstenia* based on the two rows of appendages, short ones in the inner and longer ones in the outer row. Ignoring its hanging herbaceous habit, the specimens keyed out closest to *Dorstenia benguellensis* Welw. in the key to “Succulent and semi-succulent species of the Old World” (Bergman and Hijman 1999). They also mention in the species description that *Dorstenia benguellensis* Welw. grows “often among rocks” close to water courses and that its morphology is highly variable.

Therefore in this study, I compared the specimens collected from the Luama Wildlife Reserve with *Dorstenia benguellensis* Welw. to determine: 1) whether differences are insignificant and the existing description of *Dorstenia benguellensis* Welw. should be broadened to incorporate these specimens, or 2) whether differences are significant and these specimens should be described as a separate species. I argue that differences beyond habit are significant and that they merit their own status as a new species from the Luama Wildlife Reserve, *Dorstenia luamensis* M.E.Leal.

## Methods

The collected specimens from the Luama Wildlife Reserve were compared to *Dorstenia benguellensis* Welw. following terminology and description format of Berg and Hijman (1999). Similarly, characteristics used in the Berg and Hijman (1999) key were applied to validate whether these specimens from the Luama Wildlife Reserve were significantly different from similar species within section *Kosaria*. The website Global Plants (www.plants.jstor.org) was also consulted to identify and measure specimens of *Dorstenia* collected and entered into the database after the publication of Berg and Hijman (1999).

## Results

Table 1 shows the description of *Dorstenia benguellensis* Welw. and the specimens collected in the Luama Wildlife Reserve. Besides the hanging habit and lack of latex, the specimens collected differ most distinctively from *Dorstenia benguellensis* Welw. on leaf arrangement, spiral versus horizontal; leaf shape subfalcate with an asymmetric base versus oblong, sub(ob)ovate, linear, elliptic, ovate and a symmetrical base; and peduncle length 0.1 cm versus (0.3)0.5–2.5(7) cm. The only specimen of *Dorstenia benguellensis* Welw. entered in the Global Plants database website collected close to a waterfall was M.G. Bingham 13204, but it did not resemble the specimens collected at the waterfall in the Luama Wildlife Reserve as its habit was erect and its leaves ovate.

**Table 1.** Comparison *Dorstenia benguellensis* and *Dorstenia luamensis*.

	*Dorstenia benguellensis*	*Dorstenia luamensis*
Plant	Succulent herb	Herbaceous Herb
length	Up to 50(-60) cm	10–17 cm
Root system	Tuber	Tuber
posture	Erect	Hanging
Leaves arrangement	Spiral	horizontal
lamina	Oblong, sub(ob)ovate, linear, elliptic, ovate	Subfalcate
dimensions	1–15×0.2–4.5 cm	5–7×1.1.4 cm
apex	acute to subacuminate or obtuse	Acute and micrunate
base	cuneate, sometimes obtuse to rounded	asymetrical cunate and rounded
margin	finely to rather coarsely dendate (to subcrenate) or sometimes serrulate	Entire to coarsely dendate
surfaces	puberulous to hirstellous or to hispidulous	Glabrous, subspiculate, bicolorous
lateral veins	4–12, up to 25 pairs, often (fainly) loop connected, reticulum rather narrow	5–7
petiole	(0-)0.1–0.2(-0.5) cm long	1–2 mm long
stipules	persistent, triangular to oblong, up to 5 mm long, sometimes foliaceous, puberulous	Not observed
Inflorescences	solitary or sometimes in pairs	solitary
Peduncle	(0.3-)0.5–2.5(-7) cm long, ca. 1–1.5 mm thick	0.1 cm long
	minutely puberulous to hirstellous or to hispulous	glabrous
receptacle	discoid to broadly turbinate, sometimes, shallowly cup-shaped, suborbicular 0.5–2(2.5) cm in diameter	elliptic to round, 3–4 mm in diameter, patelliform
	outside sparsely, to densely minutely puberulous to hirtellous to hispidulous	glabrous
flowering face	(sub)orbicular, sometimes to subangular or almost elliptic, fringe up to 1 mm broad or absent	elliptic to round
appendages	inner (=marginal) row numerous, triangular to subulate or filiform, up to 1–5(-7) mm long, forming a (sub)crenate rim, or indistinct and the rim entire to faintly repand,	triangular lobes, 1 mm
	outer (=submarginal) row, usually ca. 5–12 mm, less commonly more than 12, up to 23, or less than 5, down to 2, or even 0, (broadly) ligulate to filiform or sometimes subspatulate or oblong, (0.1)0.2–3.5(-8) cm long, up to 2.5 mm broad	subspathulate, 2–3 mm long, 0.5 mm broad
staminate flowers	±crowed, tepals 2, puberulous with white, red-brown or almost black hairs, stamen 2, filaments ca. 0.3–0.5 mm long, slender	few, glabrous, stamen 2, filaments ca.0.1 mm
pistillate flowers	several to many, free part of the perianth shortly tubular, puberulous with white, red-brown or almost black hairs, stigmas 2, filiform, ca. 0.2–0.3 mm long, equal or unequal in length, sometimes one of the stigmas strongly reduced or a single stigma	few, glabrous, stigmas 2, filiform, ca. 0.1 mm long,
Endocarp	body tetrahedral to subglobose, ca. 2 mm long, tuberculate, pale brown	Not observed

## Discussion

*Dorstenia benguellensis* Welw. and the specimens collected from the Luama Wildlife Reserve are most conspicuously different in vegetative morphology, both in size and shapes. Key differences mentioned in the key to “succulent and semi-succulent species of the Old world” (Berg and Hijman 1999) distinguishes sister species mainly based on vegetative morphology, e.g. absent, short or long internodes; habit hanging, erect or ascending; root tuberous or rhizome at the base and or at internods; petiole short or long; number of lateral veins; length of the plant; and some ambiguous characteristics for some species such as absence or presence of stipules.

*Dorstenia benguellensis* has been characterized as highly variable, which might raise the question whether the specimens from the Luama Wildlife Reserve are an adaptation to growing on vertical rock faces. Are there two types of *Dorstenia benguellensis* Welw.? The most common one is erect and grows in between rocks and the hanging one is rare and only grows on vertical rock faces. If this were the case, than I would have expected to find the erect type at the same location of the hanging type. This was however, not the case.

## Conclusion

Based on the differences in vegetative morphology, the specimens from the Luama Wildlife Reserve can easily be keyed out from the sister species in the same section of *Kosaria* (see the key provided under “distinct from other species”. These plants from the Luama Wildlife Reserve resemble ferns hanging from rocks. This has not been observed elsewhere for the genus. Therefore, I conclude that the specimens from the Luama Wildlife Reserve merit their own separate status as new species, *Dorstenia luamensis* M.E.Leal, sp. nov.

## Taxonomic treatment

### Dorstenia luamensis

Taxon classificationPlantaeRosalesMoraceae

M. E. Leal sp. nov.

urn:lsid:ipni.org:names:77142870-1

Figs 1A, B , 2

#### Diagnosis.

Haec species notabilis ab omnibus *Dorstenia* speciebus differt ob filicinu lithophitu habitu novu familae

#### Type.

The Democratic Republic of Congo, Katanga Province, Tumbwe Sector, Luama Wildlife Reserve, M.E. Leal 2551 (holo LWI, iso BR), S5°14,526', E 28° 52,215', 1176m, 31 October 2012.

#### Description.

Lithophytes 10–17 cm long with a tuber 0.5 cm; stems aerial, hanging, glabrous; internodes 2.5–3 cm long; no white latex or translucent exudate. Stipules absent or deciduous without scars. Leaves distichous; blade narrowly subfalcate 5–7 × 1–1.4 cm, membranaceous, apex micrunate, base cunate, adaxial side glabrous and subspiculate, abaxial side white and glabrous; margins entire; petiole 1–2 mm long; venation brochidodromous; 5–7 pairs of secondary veins; tertiary veins scalariform. Receptacle elliptic to round, 3–4 mm in diameter, patelliform; margin greenish with triangular lobes (1 mm) and subspathulate appendages, 2–3 mm long; peduncle 1 mm long, glabrous. Staminate and pistilate flowers (7 to 8) tightly packed in receptacle: perianth short lobed, whit apex minutely 2–3 lobed, glabrous; stigma 0.1 mm long. Drupes and seeds are unknown.

**Figure 1.**
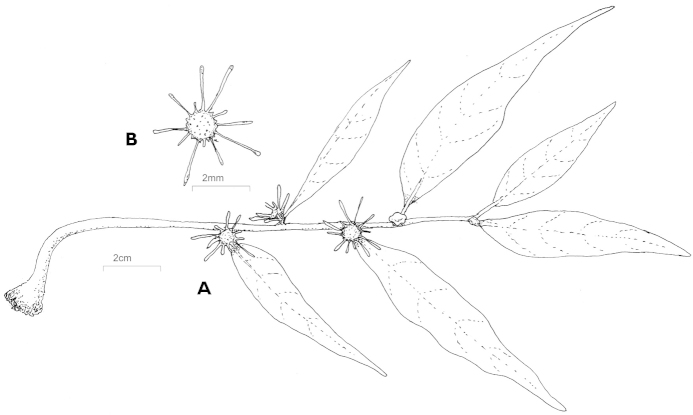
*Dorstenia luamensis* M.E. Leal **A** habit **B** receptacle.

**Figure 2.**
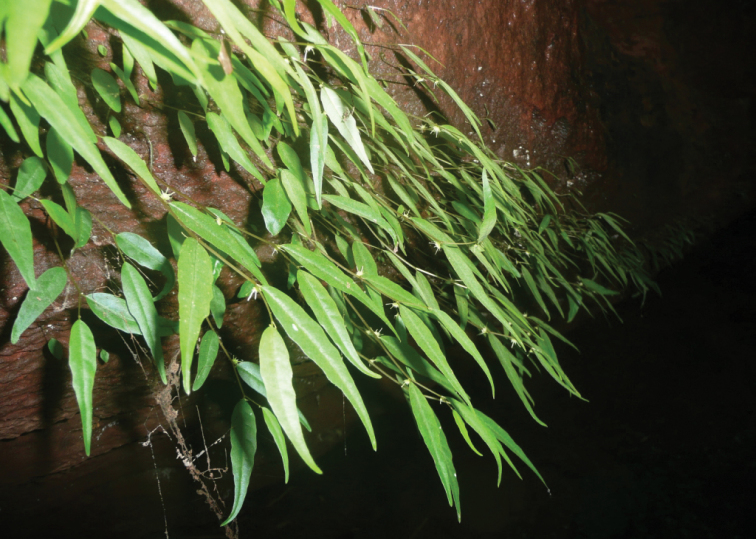
A population of *Dorstenia luamensis* M.E.Leal on a vertical rock face (photo: M.E. Leal 2012).

#### Distinction from other species.

This new species can be distinguished from any other *Dorstenia* species by its fernlike habit, hanging from vertical rock faces and the absence of latex.

The new species is added to the existing key of Berg and Hijman (1999) the “key to succulent and semi-succulent species of the Old World”

**Table:** 

1	Stems succulent and thick, internodes short; leaves subrosulate	**section *Acauloma***
1’	Stems (semi-)succulent, or herbaceous, internodes long; leaves spaced, sometimes crowded at stem apices	**section *Kosaria*, 2**
2	Plants stem hanging	
3	Plant lithophyte, multiple appendages	***Dorstenia luamensis***
3’	Plant epiphyte, one appendage	***Dorstenia astyanactis***
2’	Plants stem erect to ascending	
4	Plants annual, without a rhizome or a tuber	***Dorstenia annua***
4’	Plants perennial, with rhizome or a tuber	
5	Petiole relatively short, (0-)0.1–0.2(-0.5) cm long	***Dorstenia benguellensis***
5’	Petiole relatively long (0.2-)0.5–2.5(-3) cm long	**other species of section *Kosaria***

#### Phenology.

The specimens were collected in late October.

#### Ecology.

*Dorstenia luamensis* M.E.Leal inhabits moist and shady vertical rock faces close to small waterfalls in forest within a riverine forest-open woodland-savanna mosaic.

#### Distribution and conservation status.

The species is distributed in small populations within the type locality, and according to these demographic characteristics it merits the conservation status of endangered (EN).

#### Etymology.

The epithet *luamensis* refers the Luama Wildlife Reserve which is drained by the Luama River.

## Supplementary Material

XML Treatment for Dorstenia luamensis
